# Water use and productivity of *Cannabis sativa* L., KwaZulu-Natal Midlands, South Africa

**DOI:** 10.1186/s42238-025-00325-4

**Published:** 2025-08-29

**Authors:** G. M. Denton, A. Clulow, T. R. Hill, S. Gokool, R. Kunz

**Affiliations:** 1 Discipline of Agrometeorology, School of Agriculture and Science, University of KwaZulu-Natal, Pietermaritzburg, South Africa; 2 Discipline of Geography, School of Agriculture and Science, University of KwaZulu-Natal, Pietermaritzburg, South Africa; 3 Centre for Water Resources Research, School of Agriculture and Science, University of KwaZulu-Natal, Pietermaritzburg, 3209 South Africa

**Keywords:** Evapouration, Eddy covariance, Crop factor

## Abstract

**Aims:**

The South African National Water Act (No. 36 of 1998) mandates the regulation of land-based activities that reduce streamflow by declaring them streamflow reduction activities (SFRAs). Hemp (*Cannabis sativa* L.) is commonly known as a water-intensive crop, yet no published journal articles providing measurements of its evapotranspiration (ET) or crop factor (Kc) exist in South Africa, and there is limited information on hemp ET and Kc internationally. Therefore, its impact on streamflow reduction cannot be assessed. In the context of this research, the term water use was used synonymously with ET, and refers to the combined soil evaporation and transpiration from the *Cannabis sativa* L. crop (and when present, weeds or grasses in the interrow), which is the overall water use associated with growing the crop.

**Methods:**

This study provides ET data to determine if irrigated hemp should be investigated further as a potential SFRA by determining its ET and water productivity. An eddy covariance (EC) system was utilised in a hemp field trial. Standard microclimatic variables, volumetric soil water content, plant height, and Leaf Area Index (LAI) were measured.

**Results:**

Total ET from the hemp crop over the measurement period (7 December 2022 to 15 April 2023) was 377 mm. The average daily ET was 28.4 L/tree, or 2.94 mm/plant irrigation depth. The crop coefficient varied between 0.73 and 0.77, and the water productivity was 0.96 kg of fresh bud per m^− 3^ of water. Hemp had a high water use and low water productivity compared to international hemp studies due to a low planting density (2000 plants/ha).

**Conclusions:**

These results provide the first field-based measurements of water use and crop coefficient estimates of hemp in South Africa and contribute to the very limited data available internationally. In South Africa they will be critical to assess the streamflow reduction activity of hemp.

## Introduction

South Africa is facing a critical shortage of water, with increasing demands for the limited water resources available, with agriculture currently accounting for 66% of South Africa’s water usage (DWS, 2021). Therefore, any plans to cultivate a potentially high water-consuming crop require a detailed study of the crop’s water use, potential impacts on water resources, and downstream water availability. Failure to conduct such investigations may compromise the necessary knowledge to ensure adequate water provision for citizens and sustainable development (DWS, 2021). This scenario could develop with the increasing interest in local cultivation of *Cannabis sativa* L. due to its array of applications as a multipurpose crop within South Africa, as it can be cultivated for fibre, seed, oil, and medicinal purposes (Amaducci et al. 2015).

*Cannabis sativa* L. is grown under diverse conditions in both temperate and tropical environments (Cosentino et al. 2012; Clarke and Merlin 2016). It was declared illegal in South Africa in 1928 (Perkel 2005) however, since 2018, legislation allows for the private cultivation and consumption of small amounts of cannabis (Institute for Economic Justice 2023). Prade et al. (2011) summarised that *Cannabis sativa* L. is grown in the USA, Ireland, Spain, Germany, and Poland for biofuel purposes. *Cannabis sativa* L. is divided into two subspecies depending on their chemical composition and hence their usage. *C. sativa* L. is classified as hemp if the delta-nine-tetrahydrocannabinol (THC) content is less than 0.3%, whilst a THC percentage higher than 0.3% classifies the plant as marijuana (Wimalasiri et al. 2021). While hemp is grown for agricultural purposes (fibre and seed) and medicinal applications (cannabidiol and oil), marijuana is typically grown for personal consumption. Hemp is reported as being a high-yielding multi-purpose crop with low inputs (Struik et al. 2000). However, water and nitrogen deficiencies are two major constraints facing the hemp cultivation industry (Cosentino et al. 2013; Tang et al. 2017). Nitrogen deficiency is typically addressed through the application of appropriate fertilizers for optimum growth (Cosentino et al. 2007; Campiglia et al. 2017; Tang et al. 2017), whereas water deficiency is addressed through irrigation.

The implementation of irrigation schedules accompanying precision agriculture, requires knowledge regarding the crop water use, however, literature recommendations for the water requirements (henceforth known as evapotranspiration) of hemp are ambiguous: in a global summary provided by Pejic et al. (2018), hemp requires 250–280 mm in the Ukraine; at least 650 mm of rainfall in The Netherlands, 535 mm over a growing cycle in Tasmania, Australia, and Bocsa and Karus (1998) reported ET of up to 700 mm in eastern Europe over a growing season based on general moisture requirements in the soil and from precipitation.

A study by Cosentino et al. (2013), noted an ET of 320 mm over a full growing season of hemp in a semi-arid Mediterranean climate, while Bajić et al. (2022) observed that hemp’s ET ranged from 450 to 520 mm over a two-year period, across three hemp variants in Serbia. Both these studies applied the Class-A pan reference evaporation method with a crop coefficient (Kc) to estimate ET. However, Bajić et al. (2022) obtained Kc from Cosentino et al. (2013) who did not specify how Kc was estimated. This means that Kc cannot be validated, potentially leading to discrepancies in ET calculations for both studies. Pereira et al. (2021) reviewed recent journal papers to update FAO-56 Penman-Monteith reference evaporation (ETo) based crop coefficients, originally published by Allen et al. (1998). The authors found no papers related to Kc for hemp. Thevs and Aliev (2022) used sap flow measurements to estimate transpiration and found that hemp’s transpiration was 353 mm in northern Kazakhstan over the growing season; since sap flow measurements were spatially and temporally limited, measurement data were extrapolated to fill in data gaps. Thevs and Nowotny (2023) found that ET averaged 343 mm over a four-year trial in northern Germany using the S-SEBI remote sensing model, but lacked in-situ data and validation. However, the ET of hemp has not been derived by an internationally recognised measurement technique such as an eddy covariance system, which remains one of the most steadfast methods of ET measurement, offering non-intrusive, direct measurement at a high temporal resolution (Burba 2021). The lack of sufficient scientifically sound information on hemp ET and Kc for application with the FAO-56 Penman-Monteith ETo is particularly problematic in water-scarce countries such as South Africa (Otieno and Ochieng 2004) where there is the potential for significant expansion of the hemp industry and consequences for limited water resources.

This study measured the ET (synonymous with actual ET or water use in this research) and water productivity of hemp over a growing season in the KwaZulu-Natal Midlands, South Africa. The knowledge and recognition of hemp as a high-value crop, combined with anecdotal evidence of its successful cultivation, have led to suggestions of its significant potential for small-scale emerging farmers in rural areas of KwaZulu-Natal. However, to ensure the feasibility and sustainability of this activity, it is necessary to investigate its production potential and potential impact on water resources in areas that are already water scarce. Furthermore, the South African National Water Act (No. 36 of 1998) mandates the regulation of land-based activities that reduce streamflow by declaring them streamflow reduction activities (SFRA). While it is widely known that hemp is a water-intensive crop (Zheng et al. 2021), there is a paucity of field-based measurements of its ET both internationally and in South Africa.

## Methods

### Study site

**Fig. 1:**
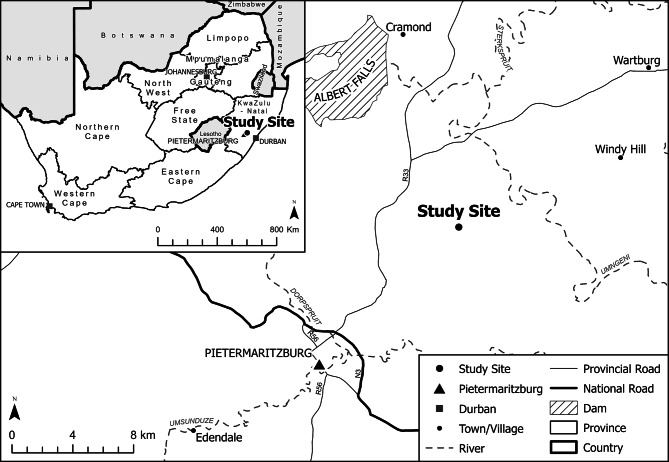
Trial site situated within KwaZulu-Natal, South Africa

The study site (29^o^31’37.0” S, 30^o^28’03.2” E) was located on a commercial farm near Pietermaritzburg in the province of KwaZulu-Natal, South Africa (Fig. 1). A seven-hectare area of *Cannabis sativa* L. was hand-planted, over a week in late November 2022, and was managed, weeded and irrigated by a local farming business. An electrified fence surrounded the site for security purposes.

The crop was partitioned into three fields, to the North, East, and South sides. The South field contained feminized *C. sativa* L. seedlings (landrace: Charlotte’s Angel), while the other two plots (North, East) contained *C. sativa* L. clones (landrace: Cherry Blossom).

Seeds and clones were germinated in a greenhouse prior to being transplanted to all three fields in November 2022. The crops were irrigated using dripper irrigation lines, with a dripper applied 0.05 m away from each plant stem to avoid stem rot. Fertigation was applied through the irrigation lines. Plants within rows were spaced approximately 2 m apart, while the row spacing was approximately 2.5 m, leading to a low planting density of 2 000 plants/ha, to mitigate the spread of disease that had occurred during previous seasons. Every fifth row was left fallow and used as a tram line for tractors to move through when spraying. In the South field, after plants had grown over 1 m tall, they were ‘topped’, which involves removing their apex to slow the plant’s vertical growth and encourage secondary branching. This is a management strategy that encourages a higher concentration of flowers to form before being harvested. The buds were harvested in April 2023.

The soils at the research site were well-drained, with approximately 74% sand, 5% silt, and 21% clay within the top 0.2 m of the soil profile, and are considered to be a sandy clay loam. A soil fertility analysis was conducted for optimal fertilizer application.

### Measurement equipment

An eddy covariance system (EC150 EasyFlux, Campbell Scientific, Logan, Utah, USA; 2) was installed in the South field on a lattice mast, positioned in consideration of the dominant wind direction to optimize the fetch, ensuring at least 100 m of hemp crop in the upwind direction, to measure total ET. The system was installed after planting on 6 December 2022 and decommissioned in May 2023, and data is presented from 7 December 2022 until 15 April 2023, when harvest occurred.

Net irradiance (CNR4, Kipp and Zonen, Delft, Netherlands) was measured on site above the canopy. To measure the flux of H_2_O over the crop canopy, an open path gas analyser (EC150, Campbell Scientific, Logan, Utah, USA) and sonic anemometer (CSAT3A, Campbell).

**Plate 1:**
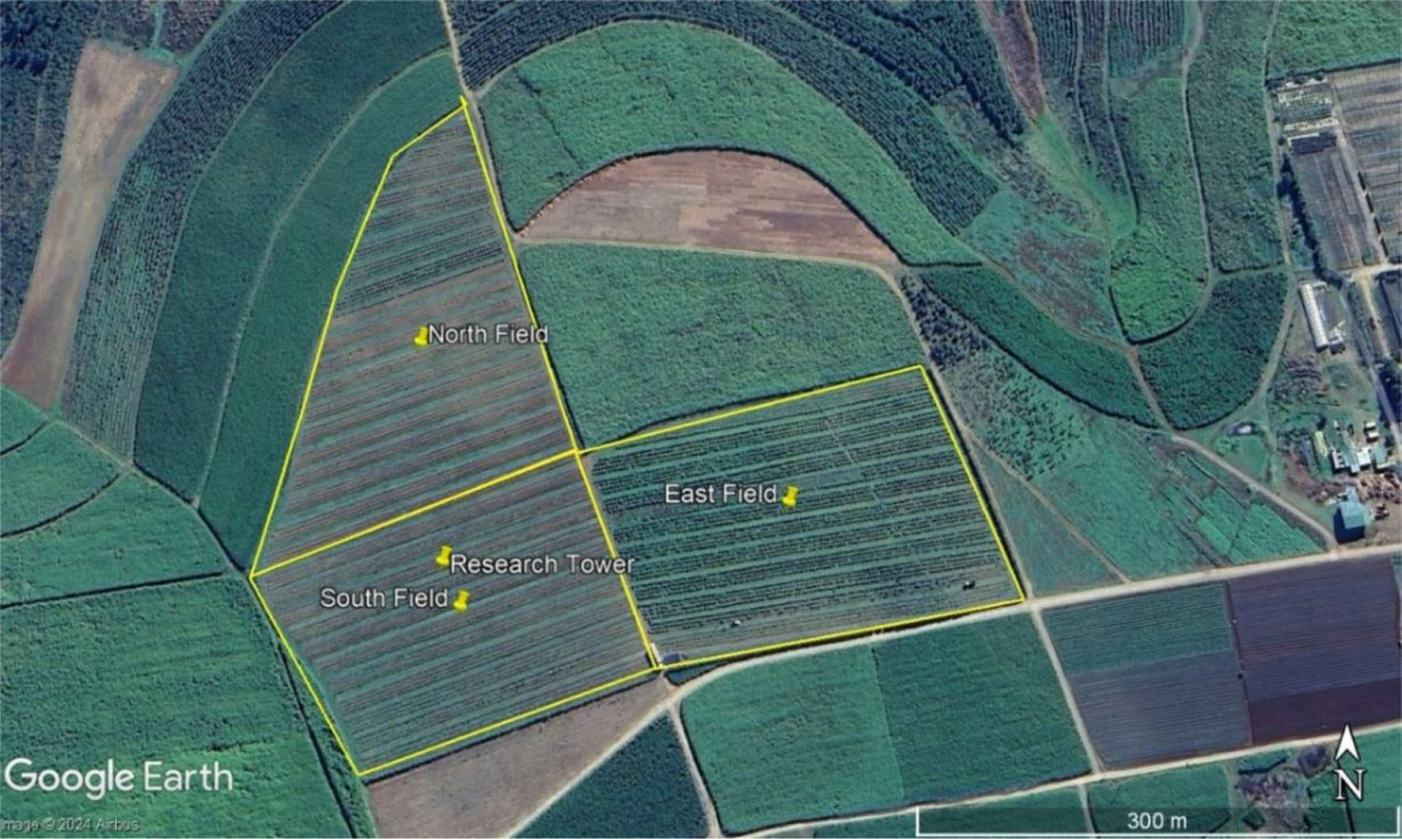
The three hemp fields and position of the measurement tower (Google Earth image, accessed 05/08/2024)

**Plate 2:**
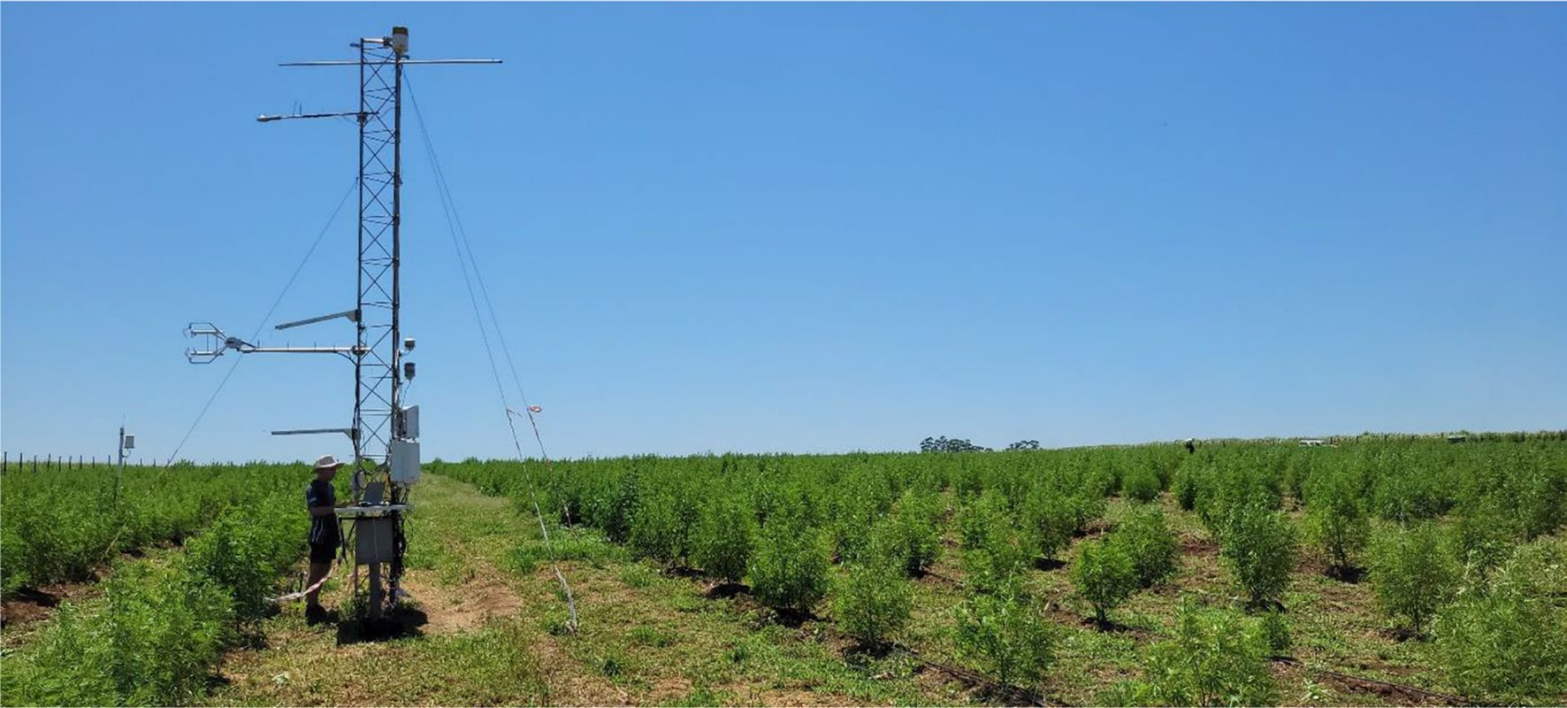
Tower and eddy covariance system measuring evapotranspiration on 24 January 2023

Scientific, Logan, Utah, USA) were used. They were attached to the mast and maintained at a height of approximately 2 m above the average crop height. Latent Energy (Eq. 1) was calculated as follows:

1 $$\:LE=\lambda\:\frac{{M}_{w}\:{M}_{a}}{\overline{P}\:}\:\overline{\rho\:}\:\overline{{w}^{{\prime\:}}{e}^{{\prime\:}}}$$

where LE was calculated by multiplying the latent heat of vaporisation (*λ*) by the ratio of molar masses of water and air, using water vapour pressure (*e*), wet air density (*ρ*), atmospheric pressure (*p*), and instantaneous wind speed (*w*). The standard coordinate rotations and corrections were applied to the datalogger (Campbell Scientific 2018).

A fine wire (FW) thermocouple (FW1 Type E, Campbell Scientific, Logan, Utah, USA) with a diameter of 25 μm was attached to the CSAT3A for high-frequency air temperature measurement. Rainfall was measured (TE525, Texas Instruments, Dallas, Texas, USA) and rainfall gaps over 3 weeks, due to a blocked rain gauge, were patched using rainfall data from a rain gauge 16 km from the site, which had a similar daily rainfall (R^2^ = 0.82). A temperature and relative humidity sensor (HC2S3, Campbell Scientific, Logan, Utah, USA) was placed in a radiation shield (41003-5, Campbell Scientific, Logan, Utah, USA) at a height of 2 m above the canopy and measured at a slow sequence of every 5 s together with the net radiation (CNR4, Kipp and Zonen, Delft, Netherlands). The EC150, FW, CSAT3A measurements were sampled at a frequency of 10 Hz using a datalogger (CR3000, Campbell Scientific, Logan, Utah, USA) and averaged at 30-min timestamps. Instruments used are summarised in Table 1.

Volumetric Water Content (VWC) of the top 0.6 m of the soil profile was measured using three water content reflectometers (CS616, Campbell Scientific, Logan, Utah, USA), which were placed within the row but away from the dripper line points. The three reflectometers were placed at depths of 0.15 m, 0.3 m, and 0.6 m.

Plant LAI was measured (LAI-2200 C, LI-COR, Inc, Nebraska, USA) by walking a straight line perpendicular to the plant rows, alternating readings between directly beneath the plants within the rows and between plants along the rows. This method was chosen to ensure full coverage of the field while minimizing labour and time constraints. Every first and fifth sample was taken above the canopy, with the second, third, and fourth samples taken below the canopy. Plant dimensions were measured using a plastic measuring pole.

### Modelling and calculations

Reference evapotranspiration was calculated using the FAO-56 Penman-Monteith method (Allen et al. 1998) with improvements by Allen et al. (2006) included. Calculations were at an hourly interval using measured net radiation, air temperature, relative humidity and wind speed from the flux tower sensors. The crop coefficient was calculated as the ratio of measured ET to ETo. Monthly Kc as well as segmented crop coefficient as described by Allen et al. (1998) are present.

The buds were harvested on 15 April 2023, and the bud mass was determined in the field before transportation to the cannabis oil extraction facility. The water productivity was determined as the ratio of wet (undried) bud mass (kg) to ET. The ET over the measurement period was converted into a volume of water (m^3^), based on the area of the field.

**Table 1:** Instruments included in the eddy covariance system

Instrument	Measurement	Manufacturer
CS616 Water Content Reflectometers	Volumetric soil water content	Campbell Scientific, Logan, Utah, USA
EC150 CO_2_/H_2_0 Open-Path Gas Analyser	CO_2_/H_2_0 flux	Campbell Scientific, Logan, Utah, USA
HFP01 Soil Heat Flux Plate	Ground heat flux	Huxaflux, Delft, Netherlands
CSAT3A Three-Dimensional Sonic Anemometer	CO_2_/H_2_0 flux	Campbell Scientific, Logan, Utah, USA
TE525mm Tipping Bucket Rain Gauge	Rainfall	Texas Instruments, Dallas, Texas, USA
HC2S3 Temperature and Relative Humidity Probe	Temperature; relative humidity	Campbell Scientific, Logan, Utah, USA
CNR4 Net Radiometer	Net solar radiation and shortwave in, shortwave out, infrared in and infrared out	Kipp and Zonen, Delft, Netherlands
FW1 Type E fine wire thermocouples	Air temperature	Campbell Scientific, Logan, Utah, USA
TCAV Type E thermocouples	Average soil temperature	Campbell Scientific, Logan, Utah, USA
LAI 2200 C Plant Canopy Analyser	Leaf Area Index	LI-COR, Inc, Nebraska, USA

## Results

**Fig. 2:**
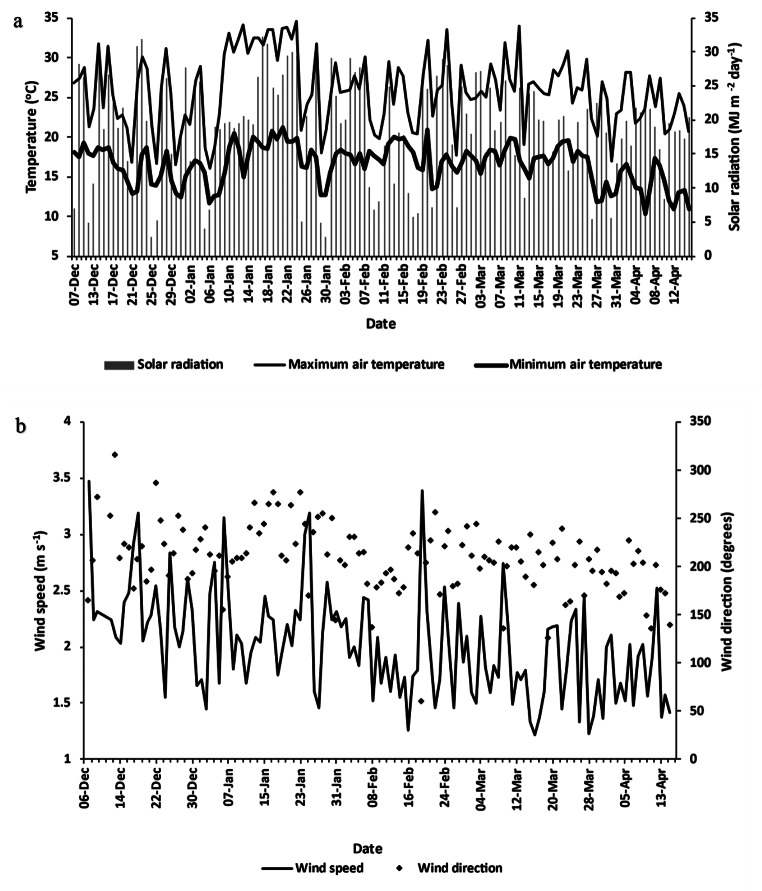
(**a**) Minimum and maximum air temperature and solar radiation; and (**b**) average daily wind speed and direction between 7 December 2022 and 15 April 2023 measured by sensors at the flux tower (Table 1)

The average temperature over the growing season was 20.3 °C (Fig. 2a), with a minimum air temperature of 10.2 °C and a maximum of 35.6^o^ C occurring due to high incoming shortwave radiation. A period of high temperatures was observed in January 2023 with maximum daily temperatures remaining above 30 °C for a two-week period. A gradual trend of decreasing minimum temperature associated with decreasing solar radiation was observed towards the end of the summer season, with April having the lowest monthly average temperature over the season (17.8 °C). Throughout the growing season, daily solar radiation averaged 13.5 MJ/m^2^/day^1^, with April having the lowest monthly average solar radiation (12.9 MJ/m^2^/day).

The average daily wind speed fluctuated between 2.2 m/s in the first half of the season and 1.8 m/s in the second half (Fig. 2b). Storms occurred on 20 February, where high wind speeds reached 3.4 m/s, with a corresponding shift in wind direction from South-South-West to East-North-East. Daily average wind speed gradually decreased from planting in late November to harvest in April, associated with a seasonal transition from mid-summer into autumn. The daily vector average wind direction was approximately 200^o^ or from the south-south-west in which there was approximately a fetch of 105 m over the hemp crop.

**Fig. 3:**
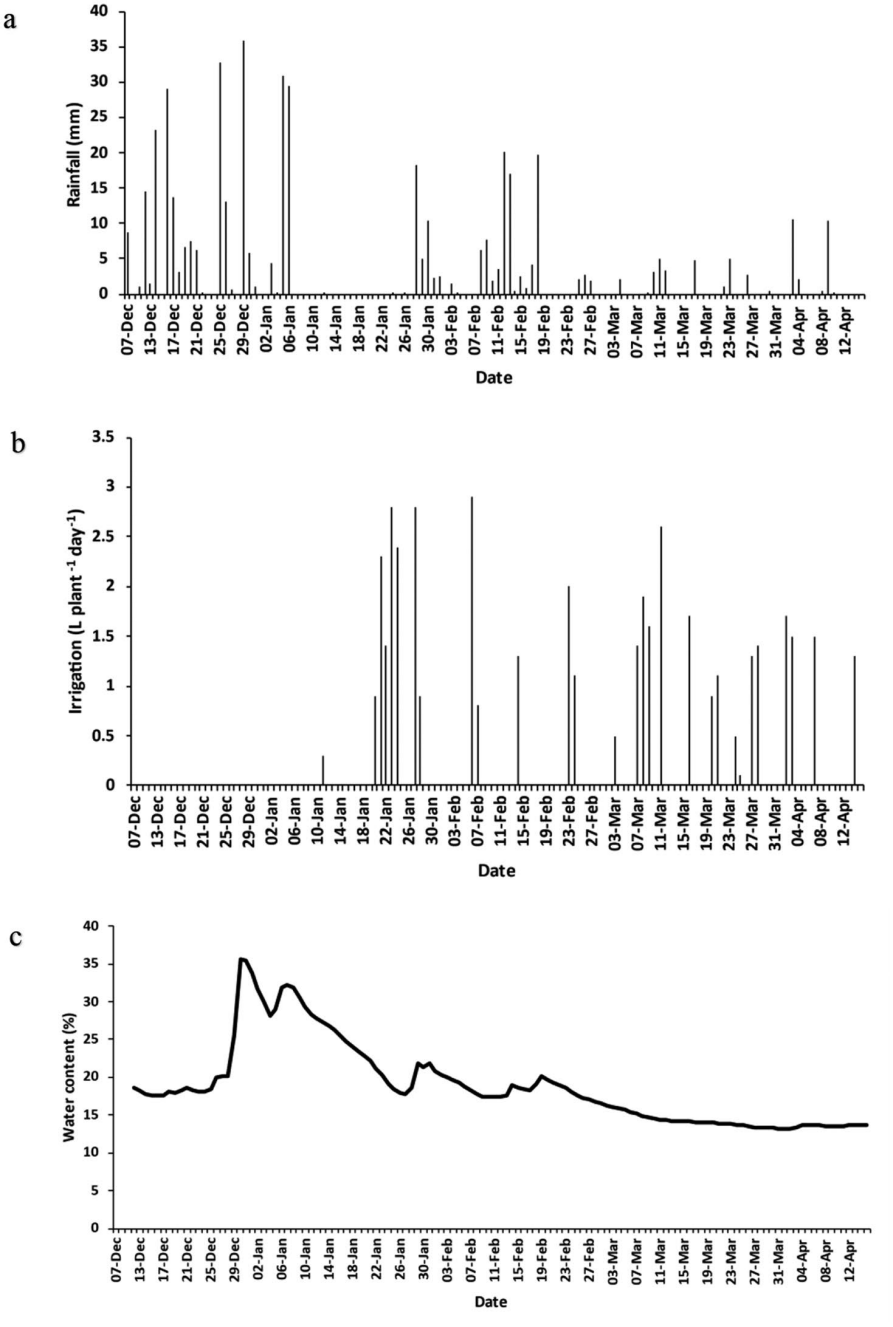
(**a**) Daily rainfall; (**b**) irrigation applied over the growing season; and (**c**) average volumetric water content throughout the soil profile between 7 December 2022 and 15 April 2023

The first six days of daily rainfall in December were not measured as the eddy covariance system was installed on 6 December 2022, yet December had the highest monthly rainfall (204 mm) over the growing season and April had the lowest (23.6 mm). As the crop was harvested halfway through the month (15 April 2023), only half a month of rainfall data was collected. The highest daily rainfall (35 mm) occurred on 29 December (Fig. 3a). A dry period was observed between 8 and 26 January where no precipitation occurred. The total rainfall over the growing season was 452 mm.

Drip irrigation was applied throughout the growing season (Fig. 3b). The soil water content was monitored using an AquaCheck soil moisture probe, and irrigation was applied accordingly. No irrigation was applied during December due to sufficient rainfall. Irrigation applied was highest in March, totalling 15 L/plant/month (1.56 mm/plant/month), followed by January (13.8 L/plant/month or 1.4 mm/plant/month), February (8.1 L/plant/month or 0.8 mm/plant/month), with April requiring the least irrigation with only 6 L/plant/month or 0.6 mm/plant/month. Conversions were based on the final plant density for the south field of 1035 plants/hectare).

The volumetric water content throughout the soil profile (measured at 0.15 m, 0.3 m and 0.6 m) was measured to represent the profile as a unit down to 0.6 m (Fig. 3c), which represents the rooting zone. The VWC fluctuated between 20% and 40%, corresponding with rainfall events, throughout the growing season. An increase in VWC (20–35%) occurred towards the end of December, due to high rainfall, before decreasing to 15%, at harvest, in April.

**Fig. 4:**
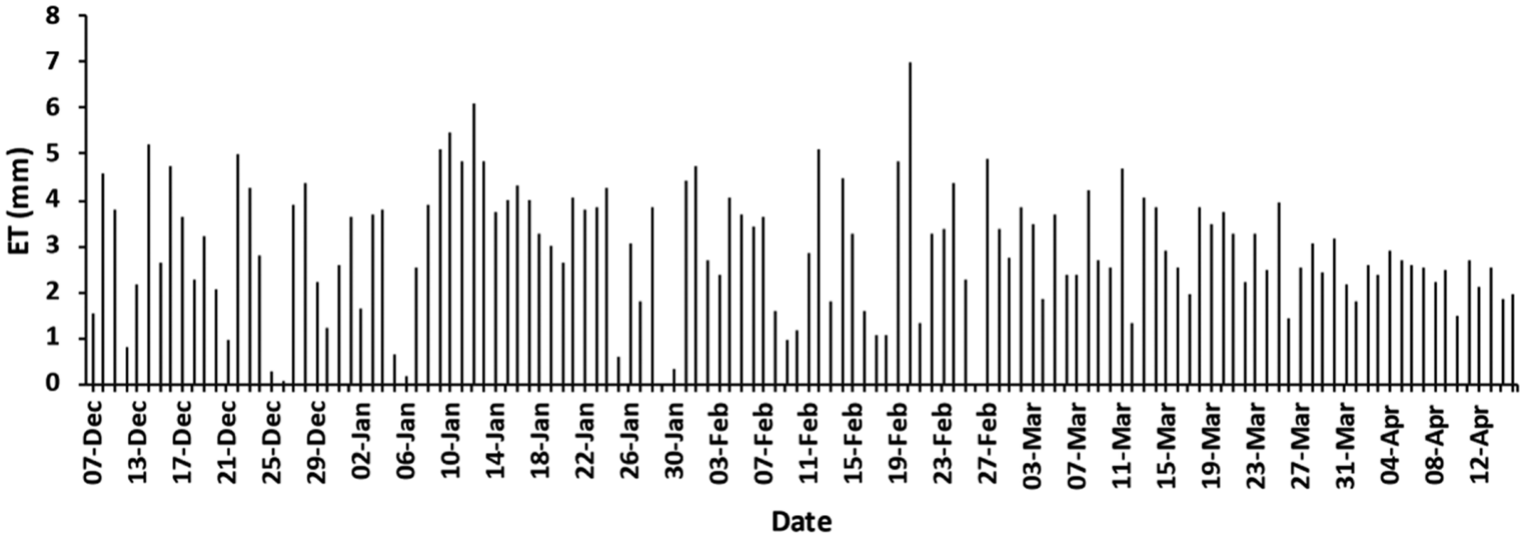
Daily evapotranspiration between 7 December 2022 and 15 April 2023, measured using eddy covariance technique

The eddy covariance system had an average energy balance closure of 87% during the day-time indicating that the EC system was working well and describing the energy balance well (Foken et al. 2010). Night-time, early morning and late afternoon data was excluded due to spikes from condensation or low fluxes. The average daily ET over the measurement period (6 December 2022 to 15 April 2023) was 2.9 mm (Fig. 4) with a maximum of 6.9 mm. High variability in ET values was observed between December and March, due to fluctuations in weather conditions, with values stabilizing and dropping as autumn progressed. Days with higher ET corresponded to hot, dry periods such as 9 to 12 January (remaining between 5 mm/day and 6 mm/day) and 20 February 2023 (7 mm/day). A summer thunderstorm occurred on 30 January with high wind speeds and hail, incurring damage, broken branches, and lodging of plants, with plant stems remaining bent over. The average water required to grow a hemp tree in the South field was 28.4 L/tree/day (2.94 mm/plant/day). This includes the water used by the weeds and that evaporates from the soil surface area around each plant which cannot be excluded from the water use of the crop in a field setting.

**Fig. 5:**
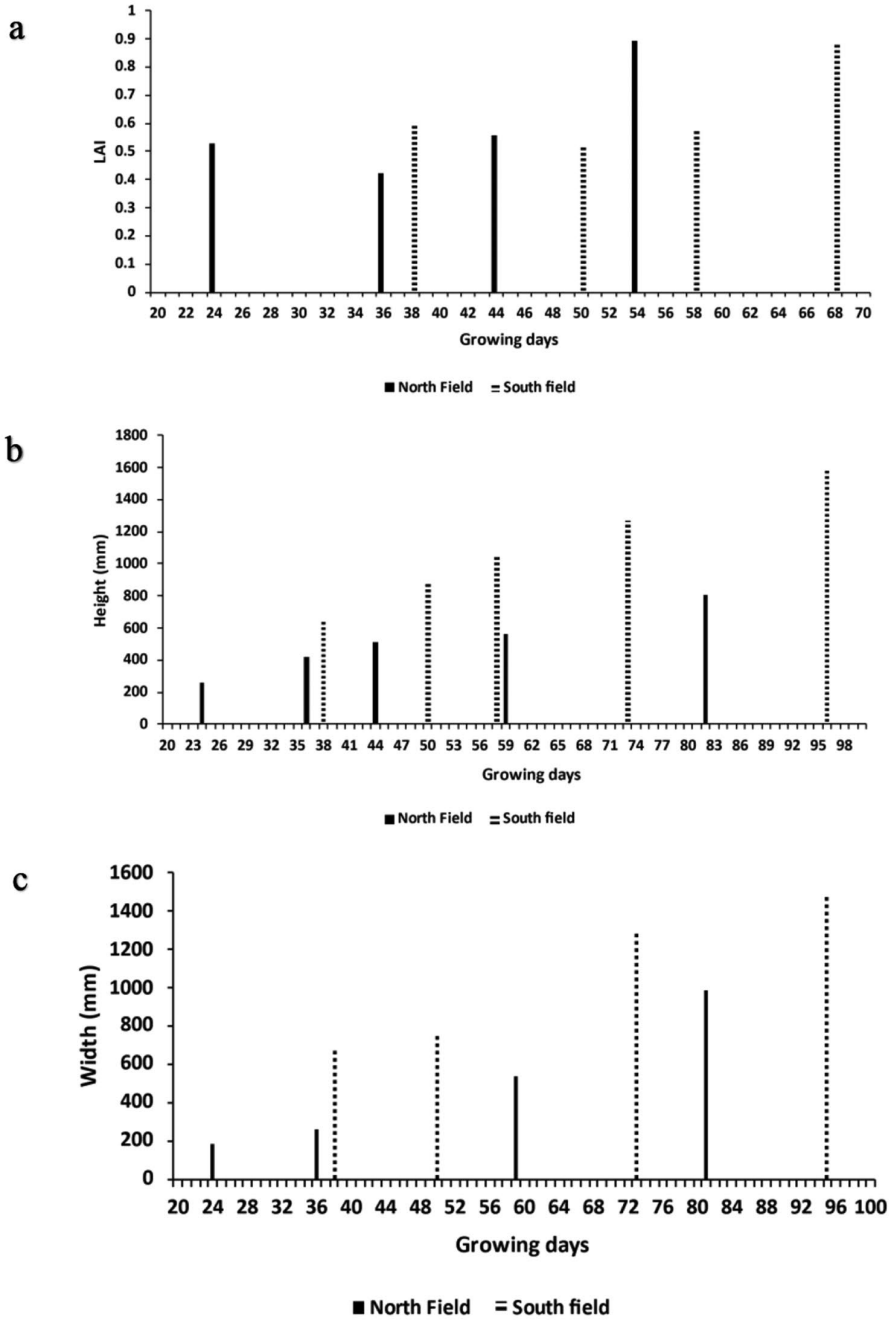
(**a**) LAI of hemp plants; (**b**) plant height of South field and North field over the growing season; and (**c**) plant width of North and South field crops over the growing season (6 December 2022 to 15 April 2023)

Due to the location of the eddy covariance system and the predominant wind direction being from the south (Fig. 2b), the area contributing to the measurements was primarily the South field. Plant dimensions and LAI were therefore recorded for the South field. However, to showcase the difference between the genetic variation of seedlings and clones, the plant diameters and LAI of the North field (clones) were included as a comparison to the South field (seedlings). The measurement of plant diameter and LAI began on growing day 24, after the seedlings had been established, however the x-axis in Fig. 5a, b and c starts from growing day 20 to provide a clearer visual representation.

In a comparison of LAI, height, and width of plant canopy between the North and South fields (Fig. 5a, b, c), the earliest measurements of LAI (Fig. 5a) included weeds, which were removed during the early growth of the crop. This leads to an overestimation of LAI-values, particularly early in the growing season. This is supported by the decrease in LAI from growing days 24 to 36 (North field), and the growing days 38 to 50 (South field). Once in the rapid growth phase, the LAI increased from 0.5 to 0.9 for both fields over a period of approximately six weeks. The South field LAI was higher than that of the North field in January (0.59 and 0.52, respectively) but slightly lower by the end of February (0.87 and 0.89, respectively).

The South field plants (seedlings) were consistently taller than the North field plants (clones; Fig. 5b), and reached a height of 1.6 m, while the North field plants reached a maximum height of 0 L.8 m at the time of last measurement. If this is extrapolated to harvest, it suggests a final height of approximately 1.0 m, still less than the canopy height of the South field plants.

The plant canopy width increased over time, with the South field plants consistently measuring higher aerial canopy cover than the North field plants (Fig. 5c). The greatest increase in plant canopy width was observed in the South field, where it grew from 0.766 m on day 50 to 1.284 m on day 73. The North field showed similar growth from 0.538 m on day 59 to 0.985 m on day 81. The final canopy diameter of the hemp canopy in the South field was 1.5 m, while the North field plants were 1.0 m.

**Fig. 6:**
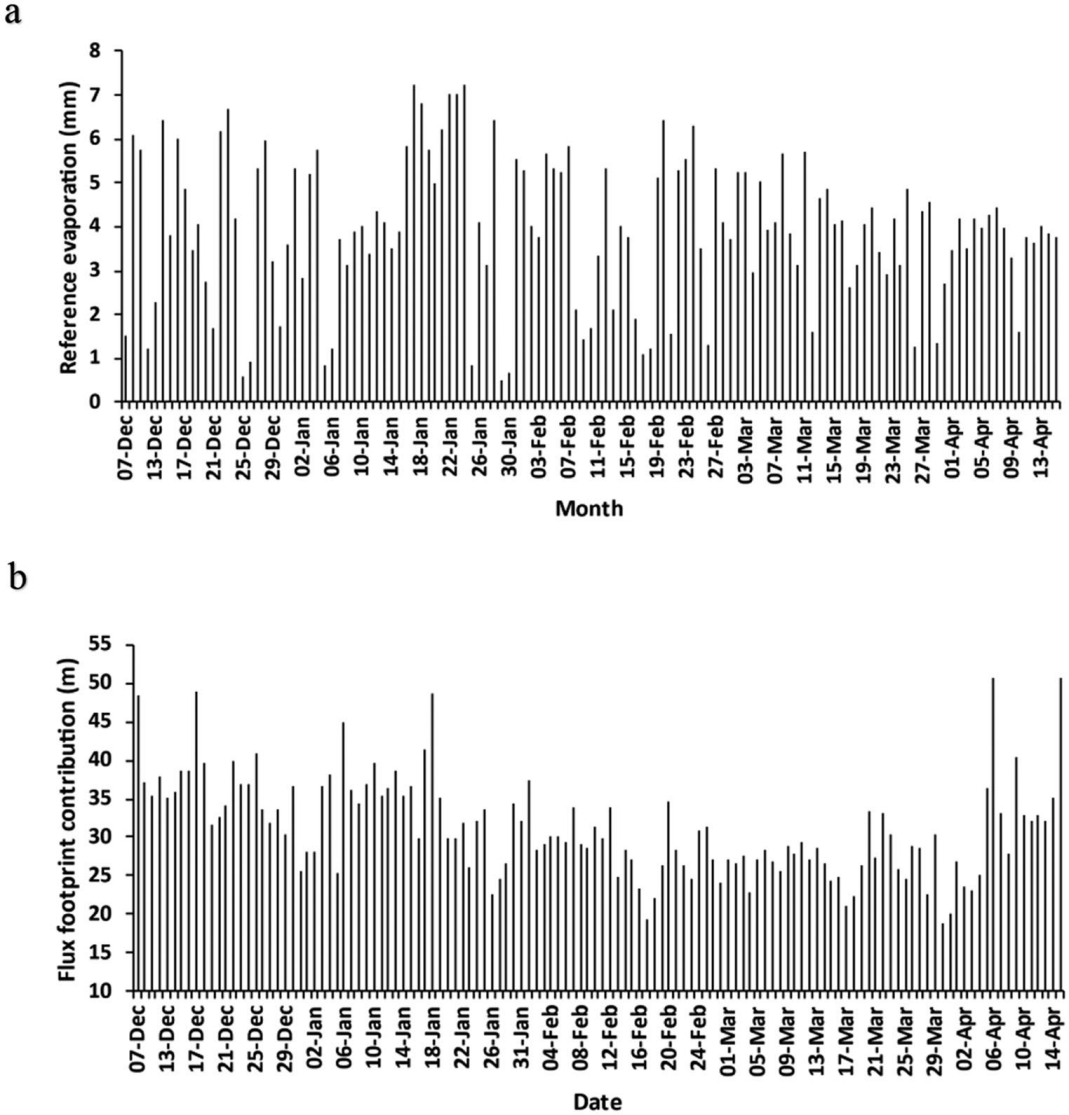
(**a**) Reference evaporation of the hemp crop between 7 December 2022 and 15 April 2023; and (**b**) distance from the research tower that had the maximum effect on evapotranspiration estimates

Reference evapotranspiration in December and January fluctuated due to variations in weather conditions in the summer rainfall area. High ETo values were observed in mid-January (Fig. 6a), peaking at 7.2 mm on 24 January. These higher values correspond with a period of clear skies (Fig. 2a) and no rainfall (Fig. 3a). Daily average ETo values were lower but more consistent during the month of April compared to December and January, due to the onset of autumn, with lower, but more consistent daily solar irradiance (Fig. 2a).

The flux footprint quantifies the surrounding area that contributes to the measurements and is influenced by numerous factors including instrument measurement height, wind speed and wind direction amongst others. It is important to quantify in order to determine that results are from the actual area of interest, namely, the Cannabis crop. The Easyflux data logger program (Campbell Scientific 2018) uses either the Kljun et al. (2004) or Kormann and Meixner (2001) models, depending on atmospheric conditions, to determine the flux footprint characteristics, including the distance upwind, where the maximum contribution to the footprint is found. The distance of maximum contribution to measurements typically remained within 20 m to 50 m from the research tower (Fig. 6b), until April 2023 where the daily flux footprint variability increased. This was in part due to raising the height of the EC system sensors at the beginning of April from 2.8 m to 3.3 m above the soil surface, to keep it approximately 2 m above the crop canopy to accommodate plant growth, as recommended by literature (Burba 2021). Although the distance of maximum contribution to flux measurements increased, there was a sufficient fetch of hemp crop to ensure that this did not affect ET measurements. The maximum distance of upwind contribution to the flux footprint indicates that the majority of the flux measurements were derived from the hemp crop or the South field.

**Fig. 7:**
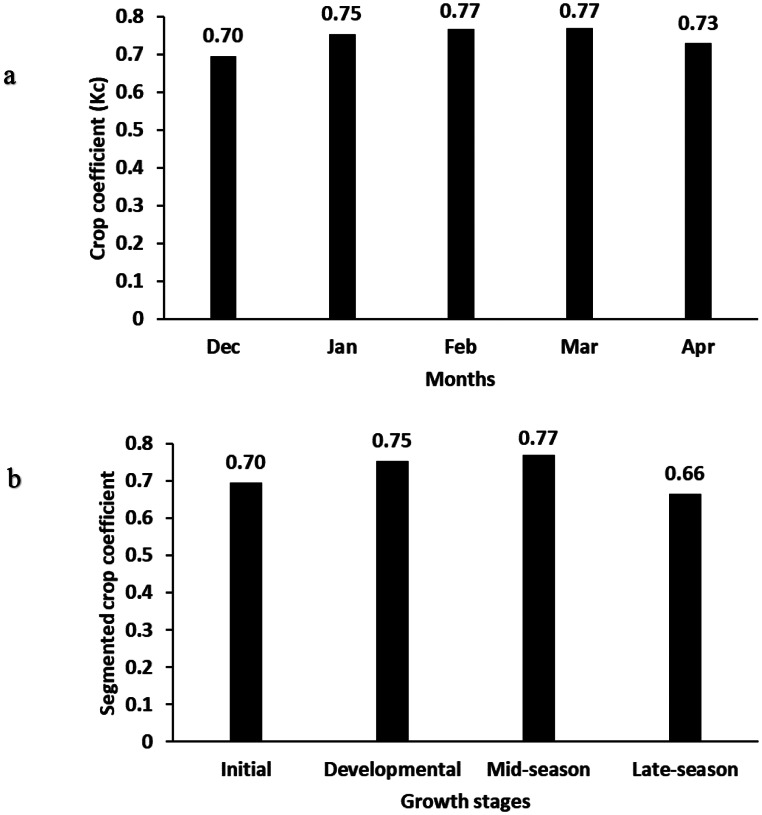
(**a**) Monthly crop coefficient of hemp; (**b**) Food and Agriculture Organisation segmented crop coefficient according to growing stage

The monthly Kc (Fig. 7a) increased from planting in December (0.70) to a peak in February and March (0.77). The Kc was lowest in April (0.73) close to harvest. Harvest took place on 15 April 2023, and as such only half a month of data was collected for April. The pattern of the segmented crop coefficient (Fig. 7b) was similar to the monthly Kc, but with a notable decline in the mature stage. It was highest during the developmental (0.75) and mid-season (0.77) stages. The initial was lower (0.70) followed by the mature stage (0.66). The average crop coefficient over the season was 0.74.

The North field, containing clones, produced a higher bud yield of 6 359 kg/ha at the time of harvest, whilst the South field bud yield was lower, at 3 623 kg/ha. The clones (North field) had a higher overall yield compared to that of the seedlings (South field) as the plant density of the South field at harvest was 1 035 plants/ha, while the North field had a harvest density of 2 000 plants/ha. Although both fields had an initial planting density of 2 000 plants/ha, the South field density was almost half that of the North field at the time of harvest due to the removal of male hemp plants to prevent cross-pollination. This significant difference in plant density, size, and yield highlights the importance of planting strategy and seed or shoot stock used in hemp farming.

**Fig. 8:**
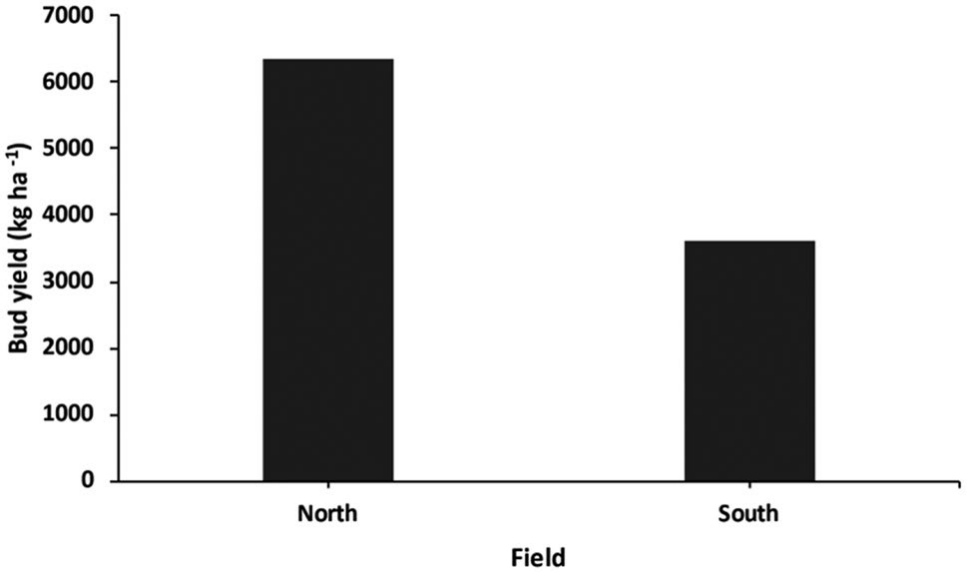
Total bud yield per hectare harvested at the end of the growing season

The water productivity (WP), which requires measurement of ET, was measured for the South field (Fig. 8), as the research tower was placed within that field, with the flux footprint obtained primarily from this field. The WP of the South field plants represents the mass of bud produced per cubic metre of water lost to ET and was found to be 0.96 kg m^− 3^.

## Discussion

The daily ET fluctuated from December until March, due to the higher variability in daily temperatures, solar irradiance, and rainfall, after which it was more stable. Comparative studies report daily water consumption as a synonym for daily ET (Cosentino et al. 2013; Thevs and Aliev 2022; Thevs and Nowotny 2023), however, Cosentino et al. (2013) calculated crop water use, rather than ET, by measuring irrigation, precipitation, and gravimetric soil water content, before and after, each irrigation event. Thevs and Aliev (2022) used sap flow measurements to calculate hemp ET, while Thevs and Nowotny (2023) used remote sensing techniques (Simplified Surface Energy Balance Index (S-SEBI) model) to estimate crop ET. The total ET of the hemp crop in the present study was 377 mm over the growing season, which is similar to that recorded by Thevs and Aliev (2022), who observed an ET of 353 mm over the growing season in northern Kazakhstan, for the hemp variety Santina 70. However, Thevs and Aliev (2022) used sap flow measurements, which measure hemp transpiration and do not include surrounding weeds, as was the case in the present study. Sap flow and EC measurements have different strengths and limitations, and neither method should be considered better than the other. For example, sap flow measurements do not account for evaporation from soil and canopy interception, and therefore do not represent actual ET (Wilson et al. 2001).

Evapotranspiration in the present study was similar to the findings of Thevs and Nowotny (2023), who observed an average of 343 mm ET over a four-year trial (April-October annually; 2018–2021) in northern Germany, with the study’s highest annual ET of 407 mm occurring in 2019. Cosentino et al. (2013) found a water use of 327 mm for the hemp variety Futura 75 in southern Italy, but this was not a direct measurement of ET. In the present study, ET of the hemp crop peaked in mid-February at 6.9 mm/day, before decreasing steadily until harvest, which is similar to Thevs and Nowotny (2023), who observed a peak ET of 6 mm/day after which ET steadily decreased until harvest. Although Cosentino et al. (2013), Thevs and Aliev (2022), and Thevs and Nowotny (2023) all utilised different approaches to the present study, they had comparable temperature regimes during the growing season (15^o^ C to 20^o^ C). However, Cosentino et al. (2013) and Thevs and Aliev (2022) both utilised drip irrigation, while Thevs and Nowotny (2023) did not apply irrigation.

Rainfall over the study site totalled 452 mm over the growing period and was higher than evapotranspiration (377 mm), with December having the highest monthly rainfall (204 mm) and April having the lowest (24 mm). According to Struik et al. (2000) the marketable value for non-irrigated hemp drops when precipitation drops below 300 mm as the plant becomes water-stressed. The period of 8 to 26 January had large rainfall gaps in which irrigation took place when required. As a result, the VWC within the top 0.6 m of the soil profile was relatively high throughout the soil profile, ensuring the prevention of water-stressed conditions. Excess rainfall at the beginning of the growth season corresponded with the increase in VWC in January (up to 35%), before maintaining a VWC of 15% between March and April. It should be noted that these results are for a well-watered crop that did not experience water stress. This was ensured through the use of an in-field, automated, AquaCheck soil moisture management system and is supported by the volumetric soil water content results of > 15% for the measurement period (Fig. 3c).

Sufficient fetch is commonly a challenge with EC measurements. The maximum flux footprint represents the distance from the research tower where the maximum influence on ET originates. The maximum flux footprint was observed to remain between 20 m and 50 m from the research tower until April 2023, where the flux footprint increased with higher daily variability occurring. There was no noticeable change in the average wind speed over this period, and therefore footprint variability is likely due, in part, to the raising of the height of the EC system sensors from 2.8 m to 3.3 m above the soil surface at the beginning of April, to keep it approximately 2 m above the crop canopy to accommodate plant growth as recommended by Burba (2021) for all crop types. Flux footprint is influenced by instrumentation height, canopy height, wind speed, friction velocity, thermal stability, surface roughness, and zero-plane displacement (Schuepp et al. 1990; Burba 2021). The maximum flux footprint extent increases with increased sensor height, while the magnitude of the peak contribution is reduced. The surface roughness coefficient would have increased as the plant height increased, and this could have affected the flux footprint.

In this research, the daily vector average wind direction was approximately 200^o^, or from the south-south-west, in which there is approximately a fetch of 105 m over the hemp crop. As a result, it was concluded that there was negligible influence of both the North and East fields (clones) on ET estimates of hemp due to the wind direction, and that the ET estimates were representative of the South field (seedlings). The daily average wind speed over the hemp crop in the first half of the growing season was 2.2 m/s, while the second half of the growing season averaged 1.8 m/s as autumn set in. Some high wind speeds were observed, and there was lodging of plants (bending of stems near ground level) and hail damage, particularly those grown from seedlings in the South field, due to their larger size.

The LAI of both the North and South fields was measured, including weeds between hemp plants. The isolation of hemp plants, and thus isolated LAI measurements, did not take place as surrounding weeds and soil surfaces are naturally found in commercial cultivation areas, adding to the water consumption of the field. The LAI was lower in comparison to previously reported values (Tang et al. 2018; Herppich et al. 2020), and declined in the early stages of the season with the North field declining from 0.52 to 0.42 between the 24th and 36th growing day, while the South field declined from 0.6 to 0.51 during the 38th to 50th growing day. This is possibly because the earliest measurements of LAI included weeds, which were removed, leading to the overestimation of initial values. The initial drop in LAI in both fields could be attributed to the removal of these weeds between the rows. The low LAI of the South field was impacted by the removal of male hemp plants, which were removed to prevent cross-pollination with the female hemp flowers and influence the chemical constituents of the bud yield (Malabadi et al. 2023). This caused the plant density to almost halve. Although the utilisation of feminized seed encourages the growth of strictly female plants, the occurrence of males is still possible.

Feminized seed is created through the application of colloidal silver or gibberellic acid to female hemp plants, inducing the formation of male flowers on these plants (Owen et al. 2023). The resulting pollination of another female hemp plant leads to the formation of seed with female chromosomes only, effectively eliminating male genetics. However, this does not guarantee a 100% success rate (Owen et al. 2023), as seen in this study. This is emphasised by the fact that only the South field, containing seedlings, had the occurrence of male hemp plants, whereas the North field, containing clones, did not. In the present study, the harvest density of the North field was 2 000 plants/ha (in-row spacing of 2 m, inter-row spacing of 2.5 m), while the harvest density of the South field was low, at 1 035 plants/ha due to the removal of male hemp plants. The North field therefore had a higher bud yield per hectare (6 359 kg/ha) than the South field (3 623 kg/ha).

Plant height and width of the South field increased quicker than the North field between January and February due to the removal of male plants, leading to less competition for light, water and nutrients and more space between plants to grow, which in turn increased the risk of lodging. Furthermore, in the present study, plants were ‘topped’ to encourage lateral growth, additional branching out of the plant and to encourage higher concentrations of flowers to form before harvest.

Water use efficiency (WUE) and WP are internationally accepted agricultural terms, yet there has been a lack of naming consistency in their usage (Sadras et al. 2012). WUE expresses the ratio at which the input of water in the agricultural system reaches the target crop, while WP expresses the water used by the crop compared to the plant biomass produced (Kilemo 2022). WUE is often incorrectly expressed as the rate of biomass production to water consumed. More specifically, the water productivity of a plant is defined as the harvested plant yield compared to the amount of water consumed (Delauney and Verma 1993; Herppich et al. 2020), or as the ratio of net CO_2_ assimilation rate to transpiration (Lamaoui et al. 2018). A review of the WP values of various crops by Zwart and Bastiaanssen (2004) indicate that it can vary, depending on factors such as climate, irrigation management and soil management. For example, Pejic et al. (2018) cited a number of global studies that indicate hemp WP values differ greatly from place to place, although the study’s sources were either not peer-reviewed or were inaccessible and could therefore not be verified. Furthermore, the few reports that do exist on the WP of hemp are difficult to compare since different bases of comparison (bark yield, biomass production, stem dry weight) were used to calculate the WP of the crop (Lisson and Mendham 1998; Di Bari et al. 2004; Cosentino et al. 2013). Previous studies have tended to focus on hemp production for different reasons, such as fibre (Cosentino et al. 2013; Tang et al. 2016, 2022), stem (Tang et al. 2017) and seed (Tang et al. 2016, 2017), whereas the present study utilised hemp bud production for medicinal purposes. WP can be increased by applying irrigation (Babaei and Ajdanian 2020), particularly in arid areas. It has been reported that hemp WP does not change under water deficit stress (Gill et al. 2022), however, this conflicts with results by Cosentino et al. (2013), who determined that the WP values of hemp, under non-stressed conditions in a semi-arid Mediterranean environment, were lower compared to the WP of the same crop under water-stressed conditions.

Cosentino et al. (2007) stated that WP values are often influenced by an over-estimation of water transpired by the crop. A study byCosentino et al. (2013) found that hemp had a WP of 2.73 kg m^− 3^ under non-water-stressed conditions in a semi-arid Mediterranean environment. In the present study the WP of 0.96 kg m^− 3^ over the growing season was significantly lower. The present study only considered the bud yield weight, whileCosentino et al. (2013) considered above-ground biomass of the whole plant at harvest. In addition, different management practices are used in the present study (such as topping), depending on the plants’ intended use. This is undesirable in bark yield analyses, biomass production and stem dry weight as it causes the hemp plants to remain shorter and shortens the length of plant fibres within the stem (van der Werf et al. 1995). The result is that WP values between studies are not always comparable. It must be noted for comparative purposes that this study measured the evapotranspiration or total evaporation (synonymous with total crop water use), which includes evaporation from the surrounding soil surface, transpiration from grasses and or weeds in the interrow as well as the Cannabis transpiration. This is justified as the presence of bare soil or interrow vegetation is unavoidable in most commercial cultivation settings (unless under certain circumstances, such as the surface being covered by plastic sheeting, which is often not cost-effective).

Many crops have prescribed Kc values, that are readily available for use with a reference evaporation (Allen et al. 1998). There are, however, only a limited number of studies that provide measured Kc values for hemp through measured reference evaporation and evapotranspiration. The following studies provide examples of where the ET of hemp was estimated using a reference (these vary) and crop coefficients, the source of which is often not provided. Cosentino et al. (2013) used a Class A pan reference in Afghanistan with segmented coefficients (source not provided) to determine irrigation requirements, Garcia Tejero et al. (2014) used the reference evaporation of Allen et al. (1998) and Doorenbos and Pruitt (1977) in Spain with average values of Kc (source not provided). Pejic et al. (2018) used the Hargreaves and Allan (2003) reference evaporation in Serbia with a Kc (source not provided), while Bajić et al. (2022) calculated hemp ET in Serbia using a Class A pan, pan coefficient and Kc (source not provided). Thevs and Nowotny (2023) derived Kc in Germany by estimating ET through remote sensing techniques and applying it to calculate ET. There is clearly a need to note the details regarding the type of reference used and origin of the Kc. Notably, two studies measure ET independently of Kc and were able to derive a Kc during the course of the study. Nougabil et al. (2019), used weighing lysimeters in Iran to measure short grass reference and hemp ET to calculate Kc, and Thevs and Aliev (2022) who measured transpiration of hemp, planted as a high growing density, using sapflow methods to calculate a transpiration crop coefficient for their own trials, located in northern Kazakhstan. The present study, therefore provides a highly sought-after monthly and segmented Kc as well as ET for a previously unresearched climatic area. Monthly values of Kc in December were 0.70, reaching 0.77 between the months of February to March, before lowering to 0.73 at harvest in April. The crop coefficient was higher than expected in the first two months of the trial when the hemp plants were small. This is likely due to the rapid growth of weeds, where weeds were bigger than the hemp plants at times, until the weeds were removed. After full development of the canopy, from February until harvest in April, the ET estimates and Kc values were more representative of the hemp than the surrounding weeds and soil surface, due to the larger size of the hemp plants than the surrounding vegetation, and the periodic removal of this vegetation. In the present study, a decline was observed in the month of April, where Kc dropped to 0.73. This is due to the onset of crop senescence. These Kc are similar to the results of Thevs and Aliev (2022), whose calculated Kc peaked at 0.7 during their growing season in northern Kazakhstan, despite the differences in the study sites.

This study determined that the overall water usage was approximately 28.4 L/plant/day (2.94 mm/plant/day). This is similar to values published by Humboldt County Outdoor Medical Cannabis Ordinance Draft, 2010, in Bauer et al. (2015) that *Cannabis sativa* L. plants use approximately 22.7 L/plant/day in northwestern California. It is further noted by Bauer et al. (2015) that ET data of hemp is limited in the published literature.

## Conclusions

There is a global interest in the expansion of areas planted under hemp, however there is relatively little information on water-use and productivity of hemp for decision makers to base their strategies of expansion upon. This is particularly important in water-deficit countries and those replacing existing food crops with hemp. This study provides the first water-use and productivity measurements of hemp grown in South Africa. The water-use over the growing season was higher than in comparative studies, and the water productivity of 0.96 kg m^− 3^ was lower than other results reported. The crop coefficient derived concurs with international studies and provides a benchmark for estimating the water use of hemp. The crop coefficient is used in hydrological models and will enable the assessment of hemp as a streamflow reduction activity in South Africa. The planting density typically used in South Africa was lower than that of international studies. This is significant as the diverse applications of the hemp plant lead to different parts of the plant being assessed in terms of WP. A further understanding of the comparison of water productivity to economic productivity, in terms of the water used relative to the economic value of yield produced in South Africa, would benefit growers.

## Data Availability

No datasets were generated or analysed during the current study.
